# A meta-analysis to study the effect of pan retinal photocoagulation on retinal nerve fiber layer thickness in diabetic retinopathy patients

**Published:** 2020

**Authors:** Meenakshi Wadhwani, Shibal Bhartiya, Ashish Upadhaya, Manika Manika

**Affiliations:** * Chacha Nehru Bal Chikitsalya, Geeta Colony, New Delhi, India; ** Glaucoma Unit, Fortis Hospital, Gurugram, India; *** All India Institute of Medical Sciences, India

**Keywords:** diabetic retinopathy, panretinal photocoagulation, retinal nerve fiber layer thickness

## Abstract

**Background.** Diabetic retinopathy is a microvascular disease, it is associated with changes in peripapillary retinal nerve fiber layer thickness, these changes being more pronounced in PDR (Proliferative diabetic retinopathy) patients undergoing laser photocoagulation.

**Objective.** To assess changes in peripapillary retinal nerve fiber layer thickness in proliferative diabetic retinopathy patients using optical coherence tomogram (OCT).

**Methods.** The database search was conducted in June 2018 and continued until October 2018. The search engines used included Pubmed, Medline, OVID and Google Scholar. A meta-analysis of weighted mean difference and standard deviation was conducted.

**Results.** A total of 10 studies containing 377 eyes of PDR patients were selected. The analysis of the included studies revealed no significant effect of PRP on average retinal nerve fiber layer thickness (0.249, 95% CI: -0.985 to 1.483) using OCT.

**Conclusion.** Hence, to conclude, our meta-analysis revealed that there was no significant effect of PRP on RNFL thickness and the impact of PRP could vary. Measurement of peripapillary RNFL thickness may yield erroneous and unpredictable results in this subgroup of patients, further confounding the evaluation of nerve fiber layer damage and its progression.

## Introduction

Diabetic retinopathy (DR) is the leading cause of blindness worldwide, especially in individuals between 20 and 65 years old. The backbone of treatment of diabetics is disability limitation, and, to an ophthalmologist, this implies prevention of the visual complications due to DR and once developed, prevention of their progression. According to the early treatment of diabetic retinopathy study (ETDRS), pan-retinal photocoagulation (PRP) is the treatment of choice for proliferative diabetic retinopathy (PDR) and severe non-proliferative diabetic retinopathy (severe NPDR). PRP reduces chances of profound vision loss by half in these types of patients [**1**-**4**]. 

There are several studies that confirmed the reduction of visual acuity even after PRP and this may be attributed to macular edema. In addition, it must be kept in mind that even though PRP mainly impacts the retinal photoreceptors and RPE, its effect may cause irreversible changes in the other inner retinal layers, as well as the RNFL.

**Mechanism of action and rationale for review**

Laser light used in PRP is primarily absorbed by melanosomes in retinal pigment epithelium (RPE), leading to photocoagulation of RPE cells and adjacent photoreceptors. As the photoreceptors are highly active metabolically, the immediate effect of this is that reduction in oxygen (O2) consumption of outer retina and diffusion of O2 from choroid to inner layers of retina occurs by creating photoreceptor free window defects and also reduction occurs in ischaemia and ischaemia induced angiogenic factors.

Although diabetic PRP helps salvage the vision, it is also associated with some grave complications in the form of higher intensity lasers leading to destruction of the entire retinal layer including ganglion cells leading to decrease in RNFL thickness.

If laser intensity is too high, it can destroy the entire retinal layer including ganglion cells, leading to a decrease in RNFL thickness. Mild retinal edema can result immediately after PRP, due to increased retinal inflammation caused by increasing vascular permeability [**5**-**10**].

DM itself can cause reduction in RNFL thickness by apoptosis of neuronal cells. In addition, decrease in retinal nerve fiber layer thickness after PRP in diabetic patients can occur due to glycosylation end products of diabetes and thermal damage from photocoagulation of retina.

The prolonged assault on the retinal nerve fiber layer thickness in patients undergoing PRP for DR means that the serial monitoring of these patients requires a careful interpretation. Any decrease in RNFL thickness in patients post PRP may be therefore due to one of four reasons:

1. Damage to retinal layer including ganglion cells due to dissipation of laser energy and consequent thermal damage;

2. Reduction in RNFL thickness by apoptosis of neuronal cells in time;

3. Reduction in RNFL thickness by apoptosis of neuronal cells as a result of continuous low-grade inflammation due to glycosylation end products of diabetes;

4. Due to comorbidity like glaucoma.

It is imperative to critically evaluate the impact of PRP on the RNFL layer in patients with diabetic retinopathy [**11**-**14**].

RNFL imaging can be performed using optic disc cube protocol that images an area of 6 mm x 6 mm with 200 x 200 scans. The inbuilt software recognizes the center of the optic nerve by utilizing a graph-based method and a circle of 3.46 mm is positioned automatically around the optic disc center to generate average and clock hour parapapillary RNFL measurements. In this meta-analysis, only the average RNFL thickness was evaluated, and only the studies with signal strength of seven or more were included for analysis [**14**-**20**]. 

## Materials and Methods

**Search Strategy**

The database search was conducted in June 2018 and continued until October 2018. The search engines used included Pubmed, Medline, OVID, and Google Scholar. The following medical subject heading (MeSH) was searched separately and then cross matched: Diabetes, Proliferative diabetic retinopathy (PDR), Pan retinal photocoagulation (PRP), average retinal nerve fiber layer thickness, Optical coherence tomogram (OCT), while limiting the search to English and human studies. From the initial MeSH searches, original articles that were published after January 2000 were analyzed.

**Study selection**

The inclusion criteria for various articles was proliferative diabetic retinopathy patients treated with pan retinal photocoagulation undergoing average retinal nerve fiber layer thickness measurement pre PRP using optical coherence tomogram and followed up at 6 months for measurement of post PRP RNFL thickness using OCT.

The exclusion criteria were: 1. Studies having no proper quantitative RNFL thickness measurement in the form of mean and standard deviation, 2. Studies without proper follow up at 6 months, 3. Studies without preoperative RNFL thickness and only postoperative RNFL thickness. 4. If the measuring tool was different from OCT. Two reviewers (M.W; M) discussed and evaluated each of the studies based on the aforementioned criteria, and any disagreement was resolved by further discussion.

**Data Extraction**

Finally, the studies retrieved were analyzed by two reviewers (MW, M) independently including first author for year of publication, location, number of subjects, type and duration of diabetes, mean age, gender, type of measuring tool, average RNFL thickness, duration of follow up.

**Quality Assessment**

The New Castle Ottawa scale (NOS) considerations were applied to each of the included studies for judging their quality. This included the criteria for subject selection, and the comparability of outcomes, as well as the case and control groups. A score of six or more indicated a study of acceptable high quality.

MW and SB carried out the assessment and to arrive at an agreement.

**Statistical Analysis**

All the studies were arranged according to the year of publication in ascending order [**21**-**30**], the mean and was recorded in the form of continuous variables, and the difference in the weighted mean and standard deviation was then calculated.

The average RNFL thickness pre and post PRP was obtained as continuous variables to calculate WMD and standard deviation. Heterogeneity of the studies was calculated by Chi square test and Higgins I2 test. The fixed effect analysis was implied if the heterogeneity was not significant (p > 0.10, I2 < 50%) otherwise random effect analysis was used. Funnel plot was also designed with the help of above data. Sensitivity analysis was performed subsequentially and one study was excluded. Then the WMD in RNFL of the remaining studies was calculated again. A p value of < 0.05 was considered significant.

## Results

**Characteristics of included studies**

The average RNFL thickness difference in diabetic patients with PDR undergoing PRP either by conventional or PASCAL PRP, before and after PRP, was analyzed using OCT. Finally, ten studies fitted the inclusion criteria. The included studies were from 2010 to 2017 (**Table 1**). 

**Table 1:** Mean and standard deviation of RNFL thickness of diabetic patients undergoing conventional PRP (pre-PRP and post-PRP) in studies using OCT

No.	Author	Year of publication	No. of eyes	Age (years)	Tool	Conventional PRP Subject's Pre-PRP av. RNFLT (Mean)	Conventional PRP Subject's Pre-PRP av. RNFLT (SD)	Conventional PRP Subject's post-PRP av. RNFLT (Mean)	Conventional PRP Subject's post-PRP av. RNFLT (SD)	Duration of FU (in months)
1	Muqit et al. [**21**]	2010	10	48 (33-62)	Stratus OCT	89.7	15.4	85.7	16.5	6
2	Kim et al. [**22**]	2013	68	59.3 ± 12.6	Stratus OCT	108.4	15	111	14	6
3	Lee et al. [**23**]	2013	31	55.2 ± 7.2	Stratus OCT	113.4	9.1	120.9	12.7	6
4	Eren et al. [**24**]	2014	58	52.4 ± 7.1	SD-OCT	108.5	17.5	103	16.5	6
5	Kim et al. [**25**]	2014	35	59 ± 8.3	Cirrus OCT	90.5	8.2	95.6	8.4	6
6	Park et al. [**26**]	2014	34	57.6 ± 10.4	Stratus OCT	111.4	2.42	110.9	2.38	6
7	Aditi et al. [**27**]	2016	27	54.3 ± 10.6	SD-OCT (Cirrus)	102	16.8	107.6	25.2	6
8	Goh et al. [**28**]	2016	39	54.97 ± 8.38	Stratus - OCT	108.8	35.3	111	30.8	6
9	Yazdani et al. [**29**]	2016	42	58 ± 6	SD OCT	90	25	88	22	6
10	Shin et al. [**30**]	2017	33	59.2 ± 10.8	Spectralis OCT	109.9	18.5	114.7	14.58	6

**Meta-analysis**

**Fig. 1:**
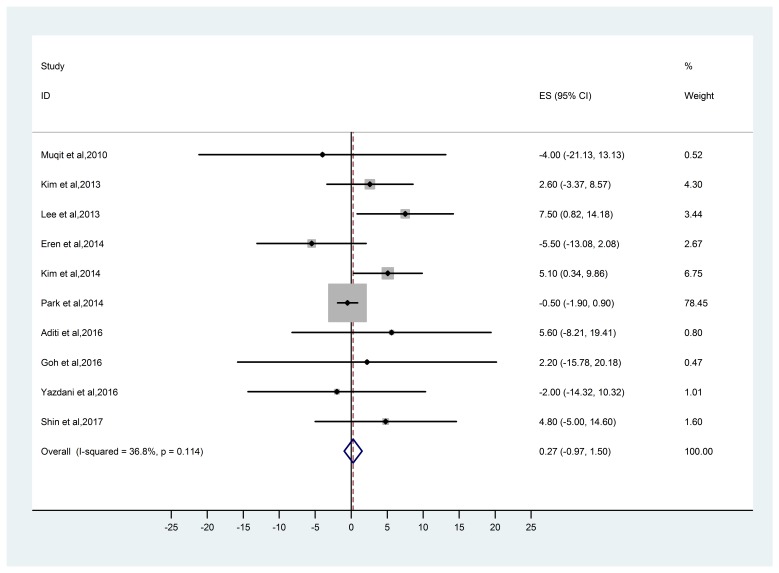
Meta-analysis of selected studies using OCT

**Fig. 2:**
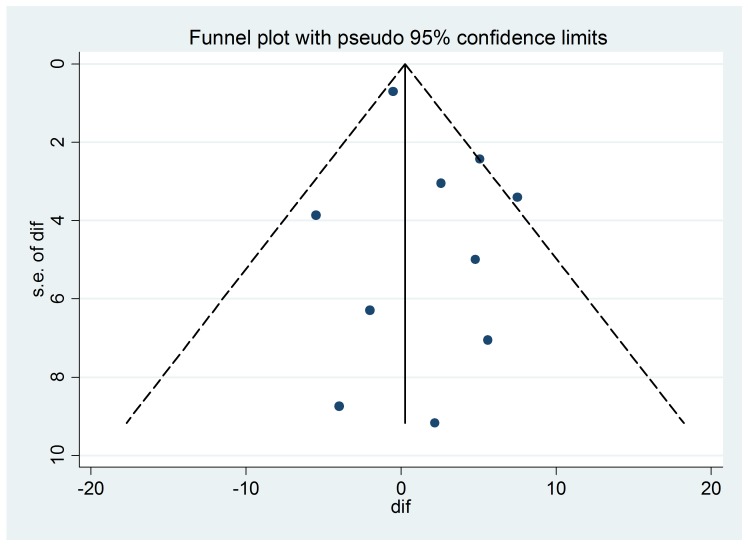
Funnel plot of selected studies using OCT

The difference in the RNFL thickness at baseline and after six months following the PRP was calculated using OCT. In the ten studies included, the heterogeneity of studies used in this meta-analysis was not found to be statistically significant (p=0.692, I2 = 30.3%). The studies revealed no significant effect of PRP on retinal nerve fibre layer thickness (0.249, 95% CI: -0.985 to 1.483) using OCT. The funnel plot suggested the absence of any obvious publication bias (**Table 2**, **Fig. 1**,**2**).

**Table 2:** Meta-analysis of average retinal nerve fiber layer thickness in different studies in diabetic retinopathy patients using OCT

Author	Effect size	95% CI		100% weight
Muqit et al. [**21**]	-4.00	-21.12	13.12	0.52
Kim et al. [**22**]	2.60	-3.37	8.57	4.30
Lee et al. [**23**]	7.50	0.82	14.17	3.44
Eren et al. [**24**]	-5.50	-13.07	2.07	2.65
Kim et al. [**25**]	5.10	0.34	9.86	6.75
Park et al. [**26**]	-0.500	-1.89	0.89	78.45
Aditi et al. [**27**]	5.60	-8.211	19.41	0.80
Goh et al. [**28**]	2.20	-15.78	20.18	0.47
Yazdani et al. [**29**]	-2.00	-14.31	10.31	1.00
Shin et al. [**30**]	4.80	-4.99	14.59	1.60

## Discussion

The purpose of the meta-analysis was to ascertain the impact of PRP on average RNFL thickness using optical coherence tomography on average retinal nerve fiber layer thickness using OCT. The OCT technology evolved from the earlier time domain system to spectral domain system. The SD OCT has been shown to produce a better scan quality, pre-perimetric RNFL defects, with better test retest reproducibility.

Various authors have used the TD-OCT to report a significant decrease in average RNFL two years after the photocoagulation [**19**,**20**]. These studies reported an initial increase in RNFL until six months after photocoagulation, with RNFL thickness measurements becoming the same as at baseline at one year. They have then reported a significant drop at two years follow-up [**16**,**17**]. Some of the newer studies have used SD-OCT for the evaluation of RNFL thickness.

Although the effect of diabetes on retinal nerve fiber layer thickness has been studied in literature in past and few studies have reported decrease in thickness of RNFL even in the absence of a history of PRP [**16**,**17**].

It is noteworthy that this observation has not been consistently reported by authors who have used the OCT to measure RNFL [**8**,**9**]. Therefore, due to such inconsistent and variable results using the same tool of OCT, we planned to perform a meta-analysis.

Authors have variably postulated that this fluctuation in RNFL thickness can be attributed to the PRP induced retinal inflammation and edema in the early post-PRP phase and subsequent degeneration of RNFL by retinal cell loss in the late–post-PRP phase. An increase in leukocyte rolling and subsequent augmentation of vascular permeability, resulting in retinal edema after PRP. On the other hand, Tso et al. have demonstrated that the high-intensity laser beam results in the damage of the entire retinal layer, including the retinal ganglion cells [**31**]. In addition, the reduction in the RNFL thickness by apoptosis of neuronal cells, and the damage due to glycosylation end products of diabetes also play a role [**32**-**35**]. The interplay of these heterogeneous factors which results in the final impact of PRP on the RNFL, in any individual patient, was possible. 

Hence, to conclude, our meta-analysis revealed that there was no significant effect of PRP on RNFL thickness and the impact of PRP could be variable. The RNFL thickness, often tends to yield erroneous and unpredictable results in this subgroup of patients, further confounding the evaluation of nerve fiber layer damage and its progression. Therefore, clinicians should keep in mind these points when interpreting the thickness in diabetics who have received PRP. 

It makes sense that diabetics undergoing PRP require more attention regarding neurodegenerative changes of the retinal nerve fiber layer and the resultant damage to vision. Multicenter studies, with a larger sample size may help understand the complexity of RNFL changes following PRP. 

**Limitation of the study**

There are certain limitations in this meta-analysis. Even though we did not limit our analysis to location or evaluation methods during our database search, we included only those studies that were in English. We did not limit our analysis to the type of OCT used, and studies using both TD- and SD-OCT have been included in the analysis. Also, we did not restrict ourselves to scanning circle of 3.4 to 3.6 mm as done in other studies, because this was not defined in the two studies. Additionally, the type of diabetes mellitus, duration of disease and glycemic control could be possible confounding factors, none of which has been considered in this analysis.

**Conflict of Interest**

The authors declare no conflict of interest.

The study was conducted according to the Declaration of Helsinki.

No funds were used to perform the study.
